# Bromelain and N-acetylcysteine inhibit proliferation and survival of gastrointestinal cancer cells *in vitro*: significance of combination therapy

**DOI:** 10.1186/s13046-014-0092-7

**Published:** 2014-11-12

**Authors:** Afshin Amini, Samar Masoumi-Moghaddam, Anahid Ehteda, David Lawson Morris

**Affiliations:** Department of Surgery, St George Hospital, 4-10 South Street, Kogarah, Sydney, NSW 2217 Australia

**Keywords:** Bromelain, N-acetylcysteine, Gastrointestinal carcinoma, Proliferation, Survival

## Abstract

**Background:**

Bromelain and N-acetylcysteine are two natural, sulfhydryl-containing compounds with good safety profiles which have been investigated for their benefits and application in health and disease for more than fifty years. As such, the potential values of these agents in cancer therapy have been variably reported in the literature. In the present study, the efficacy of bromelain and N-acetylcysteine in single agent and combination treatment of human gastrointestinal carcinoma cells was evaluated *in vitro* and the underlying mechanisms of effect were explored.

**Methods:**

The growth-inhibitory effects of bromelain and N-acetylcysteine, on their own and in combination, on a panel of human gastrointestinal carcinoma cell lines, including MKN45, KATO-III, HT29-5F12, HT29-5M21 and LS174T, were assessed by sulforhodamine B assay. Moreover, the influence of the treatment on the expression of a range of proteins involved in the regulation of cell cycle and survival was investigated by Western blot. The presence of apoptosis was also examined by TUNEL assay.

**Results:**

Bromelain and N-acetylcysteine significantly inhibited cell proliferation, more potently in combination therapy. Drug-drug interaction in combination therapy was found to be predominantly synergistic or additive. Mechanistically, apoptotic bodies were detected in treated cells by TUNEL assay. Furthermore, Western blot analysis revealed diminution of cyclins A, B and D, the emergence of immunoreactive subunits of caspase-3, caspase-7, caspase-8 and cleaved PARP, withering or cleavage of procaspase-9, overexpression of cytochrome c, reduced expression of anti-apoptotic Bcl-2 and pro-survival phospho-Akt, the emergence of the autophagosomal marker LC3-II and deregulation of other autophagy-related proteins, including Atg3, Atg5, Atg7, Atg12 and Beclin 1. These results were more prominent in combination therapy.

**Conclusion:**

We report for the first time to our knowledge the growth-inhibitory and cytotoxic effects of bromelain and N-acetylcysteine, in particular in combination, on a panel of gastrointestinal cancer cell lines with different phenotypes and characteristics. These effects apparently resulted from cell cycle arrest, apoptosis and autophagy. Towards the development of novel strategies for the enhancement of microscopic cytoreduction, our results lay the basis for further evaluation of this formulation in locoregional approaches to peritoneal surface malignancies and carcinomatosis.

## Background

Gastrointestinal malignancies account for more than one third of all deaths from cancer worldwide [1]. Representing the most common gastrointestinal cancers [2], colorectal and gastric cancers have the potential to disseminate throughout the peritoneal cavity. Although known as a manifestation of advanced disease, peritoneal carcinomatosis is a locoregional cancer spread, with the peritoneum serving as the first line of defense against the progression of carcinomatosis [3]. Hence, cytoreductive surgery in combination with hyperthermic intraperitoneal chemotherapy [4,5] has brought about long-term survival to patients with peritoneal surface malignancies [6] and is a promising approach to peritoneal carcinomatosis of gastrointestinal origin. However, disruption of the peritoneal barrier is believed to contribute to the recurrence [3]. Novel modalities are thus needed to complement the current standard of care through enhancement of microscopic cytoreduction. To this end, our research team at St George Hospital (Sydney, Australia), with an established Peritoneal Surface Malignancy Program since 1996 [7,8], has sought to develop novel locoregional approaches to peritoneal malignancies. As such, a variety of agents have been tested for their potential value in such a strategy, among which bromelain and N-acetylcysteine (NAC) as two natural agents with good safety profiles have shown promise in our investigations. We previously described the efficacy of bromelain in combination with NAC for *in situ* lysis of the mucin secreted in pseudomyxoma peritonei [9]. In the present article, we report for the first time to our knowledge that bromelain and NAC, on their own and more potently in combination, inhibit growth and proliferation of gastrointestinal cancer cells and promote cell death *in vitro*.

## Methods

### Cell culture

Human gastric carcinoma cell lines MKN45 and KATO-III were obtained from the Cancer Research Campaign Laboratories (University of Nottingham, NG7 2RD, UK) and the American Type Culture Collection (ATCC, Manassas, VA, USA), respectively. HT29-5F12 and HT29-5M21 colon adenocarcinoma cells were a kind gift from Dr Thécla Lesuffleur (Université Pierre et Marie Curie, Paris, France). LS174T colon adenocarcinoma cell line was purchased from Sigma-Aldrich (Sigma-Aldrich, MO, USA). All cell lines were maintained in a humidified atmosphere of 95% air and 5% CO_2_ at 37°C in their respective media as follows: MKN45 in RPMI-1640 medium, KATO-III in Iscove’s Dulbecco’s modified Eagle’s medium, HT29-5F12 and HT29-5M21 in Dulbecco’s modified Eagle’s medium and LS174T in Minimum Essential Medium Eagle EBSS medium (all from Invitrogen, Carlsbad, CA, USA). The culture media used were all supplemented with 10% (v/v) fetal bovine serum and 1% (v/v) penicillin-streptomycin (Invitrogen, Carlsbad, CA, USA), with the exception of Iscove’s Dulbecco’s modified Eagle’s medium being supplemented with 20% fetal bovine serum. As per the distributor’s instructions, the culture medium for LS174T was further supplemented with 2 mM Glutamine and 1% Non-Essential Amino Acids (NEAA).

### Drug preparation

Bromelain and NAC were purchased from Sigma-Aldrich (Sigma-Aldrich, MO, USA). The stock solutions were freshly made with bromelain and NAC being dissolved in relevant culture media at concentrations of 1 mg/mL and 1 M, respectively. Cisplatin was solubilized in dimethylformamide (DMF) at concentration of 0.05 M and the stock solution was stored at 4°C. Stock solutions were filtered, pH adjusted (applicable for NAC) and diluted with appropriate medium according to the final treating concentrations required for single agent and combination treatment groups.

### Sulforhodamine B assay

The effect of bromelain and NAC on growth and proliferation of the cells was determined by sulforhodamine B assay. In brief, cells were seeded into 96-well plates at densities of 1500–5000 cells/well. At desired confluence, cells were treated for 72 hours with bromelain (5–600 μg/mL) and NAC (1–100 mM), as well as with cisplatin (0.01–50 μM) as the positive control. For combination therapy, cells were individually treated with three selected concentrations of each agent and nine possible combinations of the two (Table 1). Upon completion of the treatment, cells were fixed by 30 minute incubation with 10% (w/v) trichloroacetic acid at 4°C. After five washes, plates were stained with 0.4% (w/v) sulforhodamine B (Sigma-Aldrich) dissolved in 1% acetic acid. Unbound dye was removed by rinsing the plates with 1% acetic acid and bound sulforhodamine B was then solubilized with 10 mM Tris base (Sigma-Aldrich, MO, USA). Using the PowerWaveX™ microplate scanning spectrophotometer (Bio-Tek Instruments Inc., Winooski, VT, USA), absorbance was read at the working wavelength of 570 nm.

**Table 1:** **Bromelain and NAC concentrations used in single agent and combination treatment of gastrointestinal carcinoma cells**

**Cell line**	**Single agent Bromelain (μg/mL)**							**Single agent NAC (mM)**							**Combinations (Bromelain/NAC)**								
MKN45	10	25	50	100	200	400	600	1	5	10	25	50	75	-	50/1	50/5	50/10	75/1	75/5	75/10	100/1	100/5	100/10
KATO-III	10	50	75	100	200	300	400	1	5	10	25	50	75	100	50/1	50/5	50/10	100/1	100/5	100/10	200/1	200/5	200/10
HT29-5F12	5	10	20	40	50	-	-	1	5	10	25	50	75	-	5/1	5/5	5/10	10/1	10/5	10/10	20/1	20/5	20/10
HT29-5M21	5	10	20	40	50	-	-	1	5	10	25	50	75	-	5/1	5/5	5/10	10/1	10/5	10/10	20/1	20/5	20/10
LS174T	10	20	30	40	50	-	-	2.5	5	10	20	30	40	-	10/5	10/10	10/20	20/5	20/10	20/20	30/5	30/10	30/20

### Fifty percent inhibitory concentration and drug-drug interaction analyses

Fifty percent inhibitory concentration (IC_50_) values were calculated from concentration-response curves plotting growth percentage versus drug concentration using GraphPad Prism 6 (GraphPad Software Inc., San Diego, CA, USA). The interaction between the drugs in combination treatment was determined by the median effect analysis using CalcuSyn software (Biosoft, Cambridge, UK) and the combination index (CI) was calculated based on the drug concentration and cell viability. CIs less than 0.9 and greater than 1.1 were considered as synergism and antagonism, respectively, and those between 0.9 and 1.1 as additivity.

### Western blotting

The efficacy of bromelain and NAC in inducing growth arrest and cell death was explored in MKN45, KATO-III, and LS174T cells using Western blot analysis of the relevant expressions 48 hours post-treatment. Briefly, cells were homogenized in Radio-Immunoprecipitation Assay (RIPA) lysis buffer containing 10% protease inhibitor (Sigma-Aldrich, MO, USA) and the extracted protein concentration was quantified by BioRad protein assay (Bio-Rad Laboratories, Hercules, CA, USA). After protein separation by sodium dodecyl sulfate-polyacrylamide gel electrophoresis and transfer to polyvinylidene fluoride membranes (Millipore, Bedford, MA, USA), the following primary antibodies were applied according to the manufacturers’ protocols: rabbit polyclonal anti-caspase 3, anti-Bcl2 (Santa Cruz Biotechnology, CA, USA), anti-caspase 8 (R & D Systems, MN, USA), anti-caspase 9, anti-PARP, anti-cytochrome c, anti-Akt, anti-LC3, anti-Atg3, anti-Atg5, anti-Atg7 and anti-Atg12; rabbit monoclonal anti-caspase 7, anti-Bcl-xl, anti-phospho-Akt, anti-Beclin 1, anti-cyclin B1 and anti-cyclin D2; mouse monoclonal anti-cyclin A2 and anti-cyclin E1 (Cell Signaling Technology Inc., MA, USA). A similar process was carried out for glyceraldehyde 3-phosphate dehydrogenase (GAPDH) as the loading control using the mouse monoclonal anti-GAPDH antibody (Sigma-Aldrich, MO, USA). Membranes were then washed and treated with appropriate horseradish peroxidase-conjugated secondary antibodies (Cell Signaling Technology Inc). The antigen-antibody reaction was visualized using ImageQuant™ LAS 4000 Biomolecular imager and ImageQuant™ software (GE Healthcare, Chalfont, UK).

### TdT-mediated dUTP nick-end labeling assay

The presence of apoptosis in MKN45 and LS174T cells was determined by terminal deoxynucleotidyl transferase (TdT)-mediated deoxyuridine triphosphate (dUTP) nick-end labeling (TUNEL) assay using DeadEnd™ Fluorometric TUNEL System (Promega, WI, USA) in accordance with the manufacturer’s instructions. In brief, cells were seeded onto sterile glass coverslips in 6-well plates, allowed to grow for 72 hours and then treated for 48 hours. Cells were washed twice with ice-cold phosphate buffered saline (PBS), fixed in 4% methanol-free formaldehyde in PBS for 25 minutes at 4°C and permeabilized by 0.1% Triton X-100 (Sigma-Aldrich, MO, USA) in PBS for 5 minutes (fixed cells incubated for 5 minutes with DNase I buffer and treated with 5.5–10 units/mL of DNase I (Ambion, Life Technologies, MA, USA) for 10 minutes were used as positive controls). After being washed, cells were covered with 100 μL of Equilibration Buffer for 5-10 minutes at room temperature and treated with 50 μl of recombinant terminal deoxynucleotidyl transferase (rTdT) incubation buffer at 37°C for 60 minutes inside the humidified chamber (cells incubated with an incubation buffer without rTdT enzyme were used as negative controls). The tailing reaction was then terminated by immersing the slides in 2X saline-sodium citrate (SSC) buffer for 15 minutes at room temperature. Unincorporated fluorescein-12-dUTP was removed by PBS washes and cells were stained with 1 μg/mL propidium iodide in PBS for 15 minutes at room temperature in the dark. Coverslips were washed in deionized water for 5 minutes at room temperature for a total of three times and mounted with gelatin glycerol. Cells were visualized by FluoView™ Laser Scanning confocal microscope (Olympus, Center Valley, PA, USA) and X60 oil immersion lens using a standard fluorescein filter set to view the green fluorescence of fluorescein at 520 ± 20 nm and the red fluorescence of propidium iodide at >620 nm. The FluoView™ software version 4.3 (Olympus, Center Valley, PA, USA) was used to overlay the images.

### Statistical analysis

All data presented are representative of three independent experiments. Statistical analyses were performed using GraphPad Prism 6 (GraphPad Software Inc., San Diego, CA, USA). The Student’s *t*-test was applied for unpaired samples. p values <0.05 were considered significant.

## Results

### Bromelain and NAC, on their own, significantly inhibited proliferation of the human gastric and colon carcinoma cells studied

**Figure 1:**
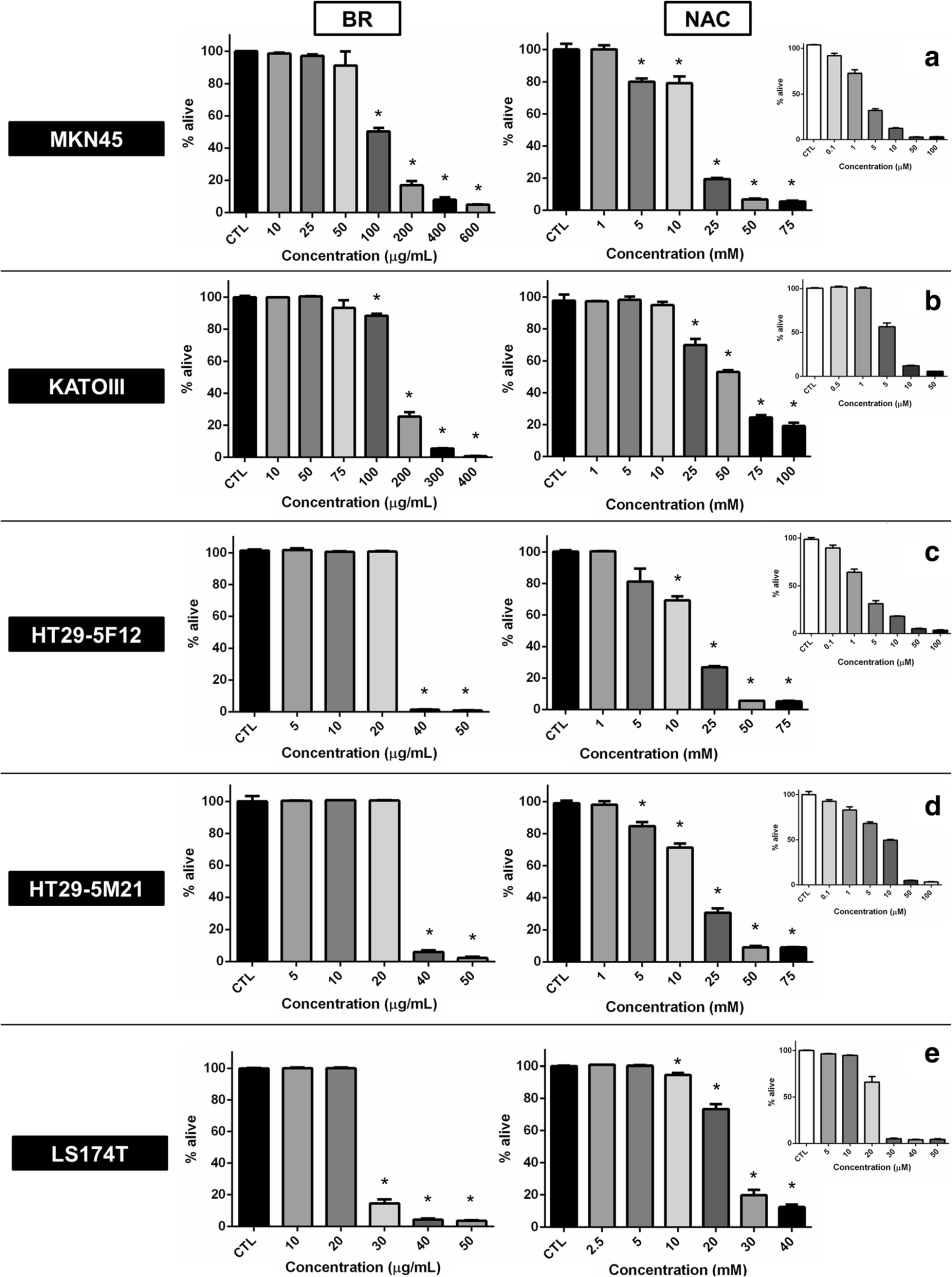
**Sulforhodamine B assay on MKN45, KATO-III, HT29-5F12, HT29-5M21 and LS174T cells after single agent treatment with bromelain and NAC.** As assayed 72 hours post treatment, concentration-dependent inhibition of cell proliferation was observed with escalating concentrations of bromelain (left panel) and NAC (right panel). Cisplatin was used as the positive control of the experiment (small graphs **a**-**e**). Significant changes (p <0.05) are marked by asterisks.

**Figure 2:**
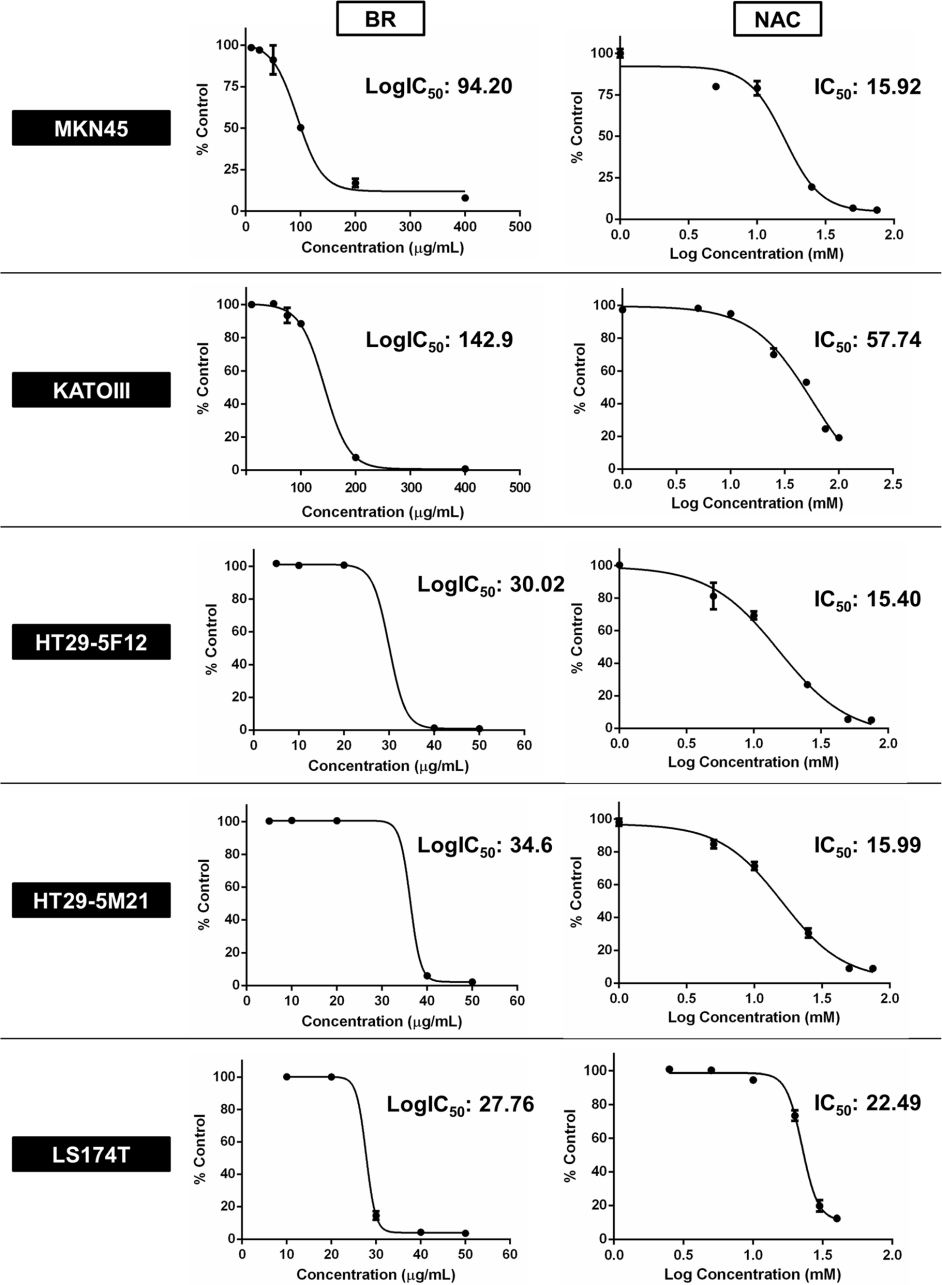
**Concentration-response curves for single agent treatment of MKN45, KATO-III, HT29-5F12, HT29-5M21 and LS174T cells with bromelain and NAC.** These curves plot growth percentage versus drug concentration after 72 h treatment of the cancer cells with bromelain (left panel) and NAC (right panel). Half maximal inhibitory concentration (IC_50_) values are demonstrated for each curve individually.

First, antiproliferative effects of bromelain and NAC in single agent therapy was evaluated by sulforhodamine B assay (Figure 1). Bromelain significantly inhibited proliferation of MKN45 (p = 0.0018, 0.0010, 0.0002 and <0.0001 for concentrations of 100, 200, 400 and 600 μg/mL, respectively), KATO-III (p <0.0001 for concentrations ≥100 μg/mL), HT29-5F12, HT29-5M21 (p <0.0001 for concentrations of 40 and 50 μg/mL) and LS174T cells (p <0.0001 for concentrations ≥30 μg/mL) in a concentration-dependent manner. Similarly, NAC exerted antiproliferative effects on MKN45 (p = 0.0006 and 0.0037 for concentrations of 5 and 10 mM, respectively, and p <0.0001 for concentrations of 25, 50 and 75 mM), KATO-III (p = 0.0071 and 0.0004 for concentrations of 25 and 50 mM, respectively, and p <0.0001 for concentrations of 75 and 100 mM), HT29-5F12 (p = 0.0003 for concentration of 10 mM and p <0.0001 for concentrations ≥25 mM), HT29-5M21 (p = 0.0091 and 0.0008 for concentrations of 5 and 10 mM, respectively, and p <0.0001 for concentrations ≥25 mM) and LS174T cells (p = 0.0026 for concentration of 10 mM and p <0.0001 for concentrations ≥20 mM) in a concentration-dependent manner. As shown in Figure 2, IC_50_ values of 94, 142, 30, 34 and 27 μg/mL for bromelain and IC_50_ values of 15, 57, 15, 16 and 22 mM for NAC were resulted from treating MKN45, KATO-III, HT29-5F12, HT29-5M21 and LS174T cells, respectively.

### Combined use of bromelain and NAC resulted in significantly more potent growth-inhibitory effects

**Figure 3:**
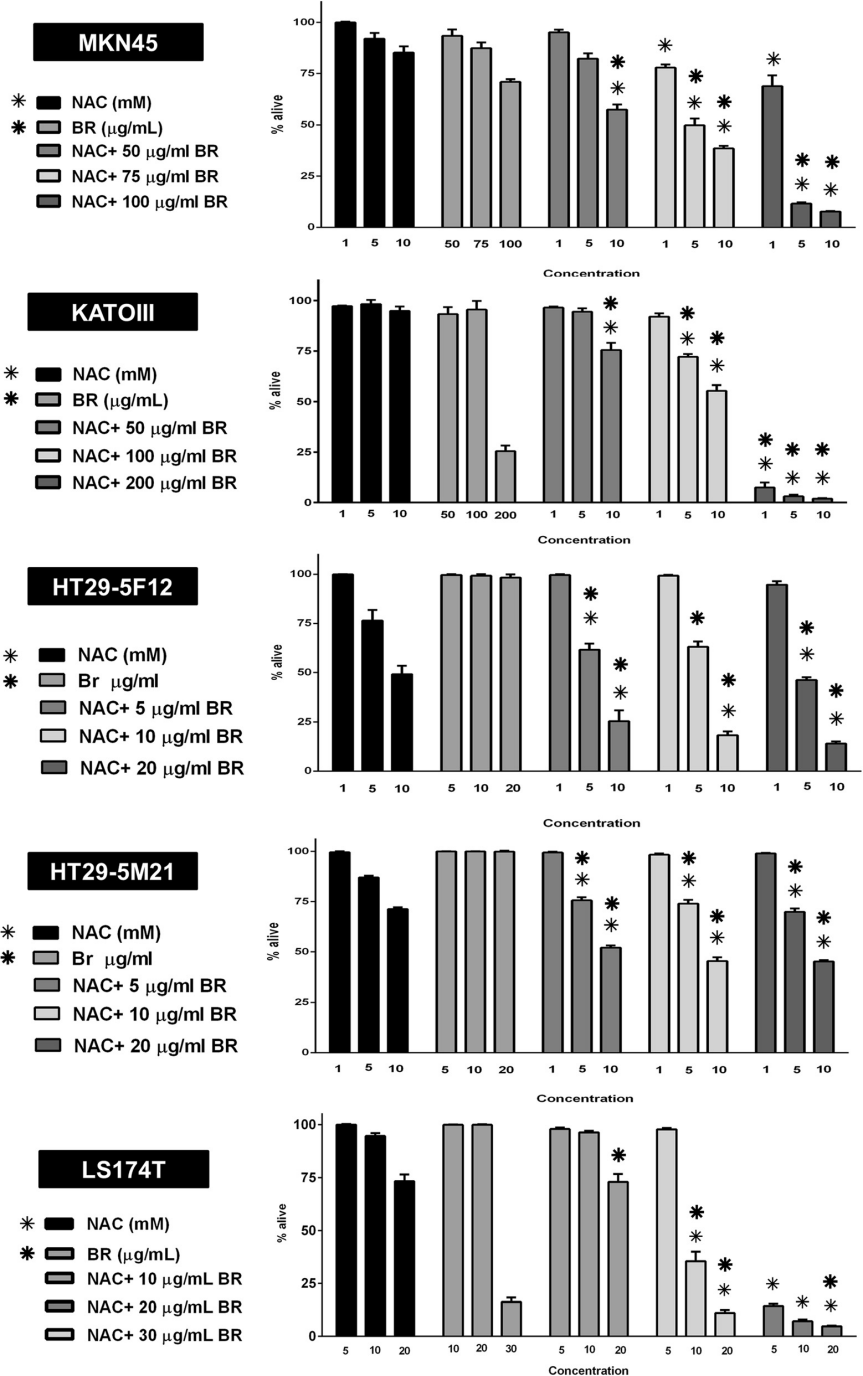
**Combination treatment of MKN45, KATO-III, HT29-5F12, HT29-5M21 and LS174T cells with bromelain and NAC.** Cells were treated for 72 hours with three concentrations of each agent on their own and in combination. In general, growth-inhibitory effects of combination therapy were significantly more potent than those induced by single agent treatment. Significant changes (p <0.05) are marked by bold (*) and non-bold (*) asterisks when single agent bromelain and NAC are considered as control, respectively.

**Figure 4:**
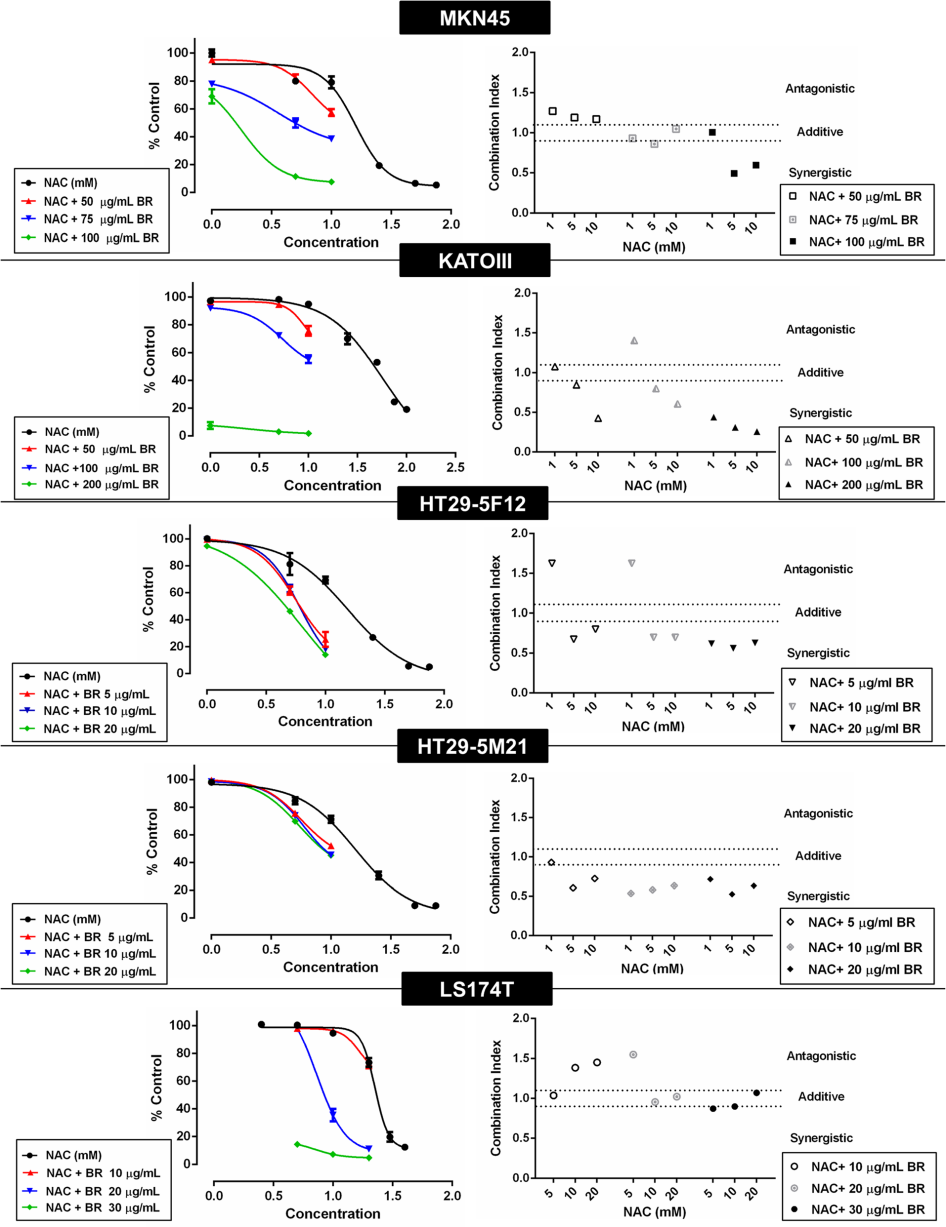
**Concentration-response curves and drug-drug interaction analysis of combination treatment of MKN45, KATO-III, HT29-5F12, HT29-5M21 and LS174T cells.** Left panel demonstrates left-sided shift of concentration-response curves of NAC after being used in combination with bromelain. Drug-drug interaction analysis (right panel) revealed synergism and additivity as the predominant patterns of interaction between bromelain and NAC in combination therapy.

Subsequently, we investigated how bromelain and NAC influence growth and proliferation of the cancer cells in combination therapy. We treated the cells with three selected concentrations of each agent individually and nine possible combinations of the two. Our data revealed that in most combination groups, growth-inhibitory effects resulted were significantly more potent than those induced by single agent bromelain and/or NAC (Figure 3). In agreement, analysis of the concentration-response curves revealed a left-sided shift in combination therapy (Figure 4, left panel).

### Synergy was the predominant pattern of bromelain-NAC interaction in combination therapy

Next, the interaction between the drugs in combination therapy was determined by the median effect analysis using the CalcuSyn software (Figure 4, right panel). In MKN45 cells, the interaction pattern changed from antagonism, observed only in the combination groups with the lowest concentration of bromelain, to additivity and synergism as the bromelain concentration increased. In KATO-III cells, synergistic effects were evident in all treatment groups, except for two groups with the lowest concentration of NAC (additive and antagonistic effects when NAC 1 mM used in combination with bromelain 50 and 100 μg/mL, respectively) whereby consistent strengthening of the synergy accompanied higher concentrations of bromelain/NAC. Apart from the antagonistic effects observed in two groups with the lowest concentration of NAC (NAC 1 mM combined with bromelain 5 and 10 μg/mL), bromelain-NAC interaction in HT29-5F12 cells was revealed to be synergistic, with a slight strengthening at higher concentrations. With no antagonism and only one additive pattern observed with the lowest concentrations of bromelain and NAC, drug-drug interaction was mainly synergistic in HT29-5M21 cells, with a fairly slight fluctuation in the potency as concentrations rose. Finally, in LS174T treatment groups, increases in the bromelain concentration were associated with resultant progress in interaction with NAC from antagonism (bromelain 10 μg/mL combined with NAC 10 and 20 mM and bromelain 20 μg/mL combined with NAC 5 mM) to additivity and slight synergism. On the other hand, with the exception of one instance (bromelain 20 μg/mL with NAC 5 mM), rises in NAC concentration resulted in the weakening of interaction with a given concentration of bromelain.

### Bromelain/NAC treatment interfered with cell cycle progression and induced cell death with involvement of both apoptotic and autophagic processes

**Figure 5:**
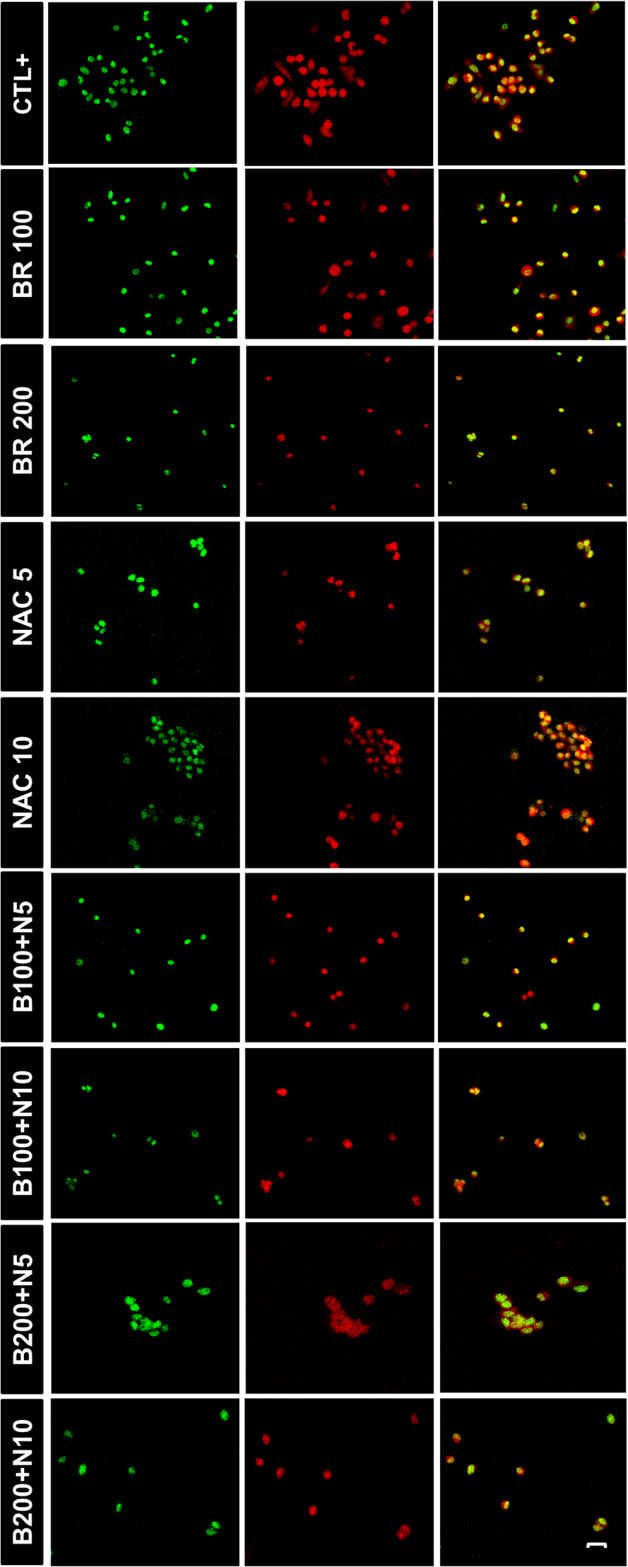
**Fluorometric TdT-mediated dUTP nick-end labeling (TUNEL) assay on treated MKN45 cells.** After 48 hours of treatment with bromelain (100 and 200 μg/mL), NAC (5 and 10 mM) or the combination, cells were assayed for TUNEL reactivity and viewed under laser scanning confocal microscope. Green (fluorescein-12-dUTP) and red (propidium iodide) fluorescence correspond to fragmented DNA and the nucleus, respectively, indicating the presence of apoptotic cells in all treatment groups as compared with DNase I-treated cells used as the positive control. Scale bar: 50 μm.

Following the efficacy study, we investigated the mechanistic basis of the growth-inhibitory effects induced by bromelain and NAC using MKN45 and LS174T cell lines. To this end, we first conducted fluorometric TdT-mediated dUTP nick-end labeling (TUNEL) assay to explore the presence of apoptosis after 48 hours of treatment with bromelain and NAC, on their own and in combination. As anticipated, apoptotic bodies were detected in both cell lines as an indication of DNA fragmentation and activated apoptotic cascades. Figure 5 depicts the results for MKN45 cells, representatively.

**Figure 6:**
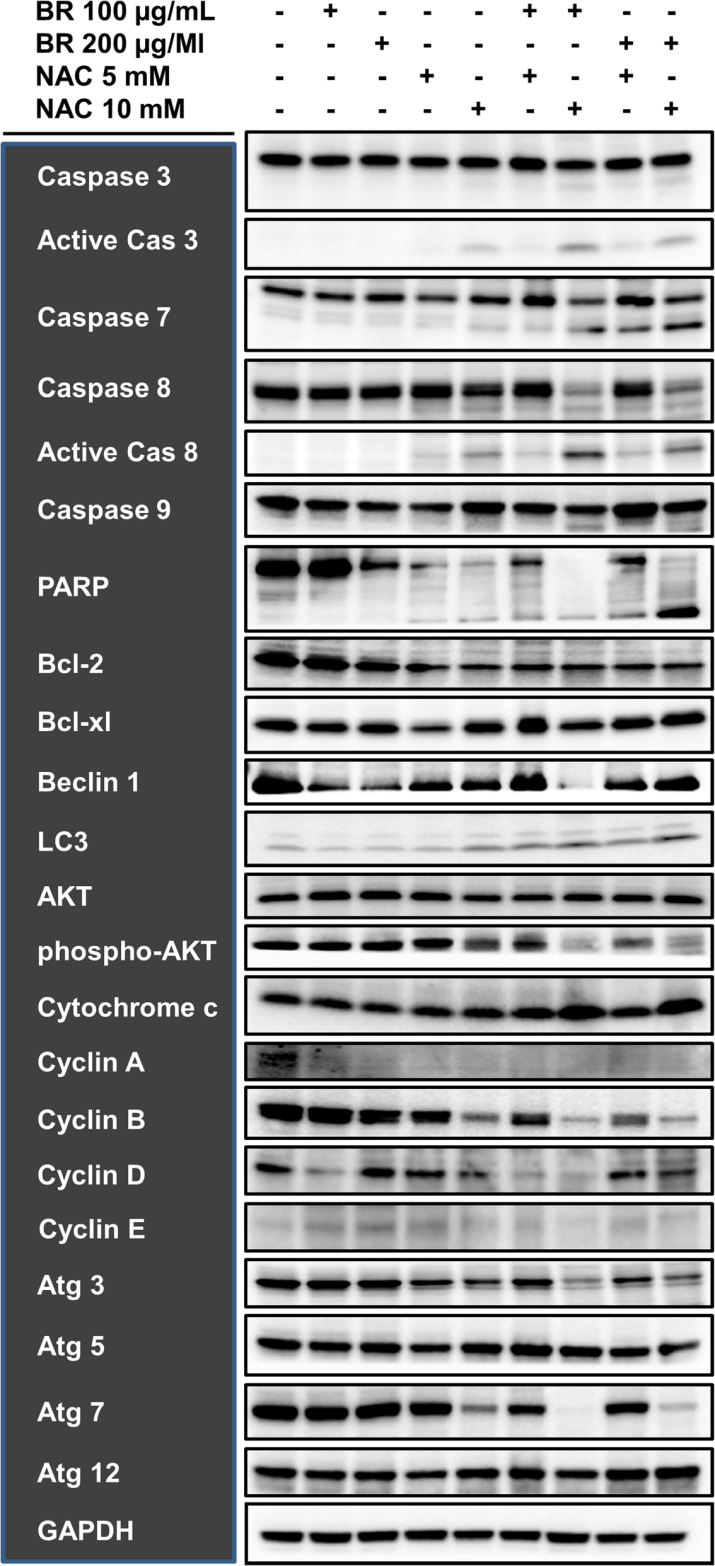
**Western blot analysis of the expression of proteins involved in the regulation of cell cycle and survival in MKN45 cells.** After 48 hours of treatment with single agent or combined bromelain (100 and 200 μg/mL) and NAC (5 and 10 mM), activation of caspase system (represented by caspase proteins, cytochrome c and PARP), inhibition of anti-apoptotic and pro-survival processes (represented by Bcl-2 and phospho-Akt), diminution of the cell cycle regulators cyclin A, cyclin B and cyclin D along with the emergence of the autophagosomal marker LC3-II and deregulation of other autophagy-related proteins, including Atg3, Atg5, Atg7, Atg12 and Beclin 1, were found. As seen, results were more prominent in combination therapy. Our data implies that growth arrest, apoptosis and autophagy are likely mechanisms underlying cytotoxic effects of bromelain and NAC on gastrointestinal cancer cells. GAPDH was used as the loading control.

To further explore the underlying mechanisms of effect, we then analyzed the expression of a variety of proteins involved in the regulation of cell growth and vitality in MKN45, KATO-III and LS174T cells after single agent and combination therapy with bromelain and NAC. For this purpose, Western blot analysis was performed after 48 hours on treated cells as well as on their untreated counterparts as the negative control. As apparent from MKN45 blots shown as our representative results in Figure 6, treatment with bromelain and NAC appeared to inhibit cell survival and to induce apoptotic and autophagic cell death while interfering with cell cycle progression. Firstly, the expression of the cell cycle regulatory proteins cyclin A, cyclin B and cyclin D was found to be diminished in treated cells. Secondly, activation of caspase system was evident as the emergence of immunoreactive subunits of caspase-3, caspase-7 and caspase-8, withering or cleavage of procaspase-9, and overexpression of cytochrome c. The functionality of activated caspase-3 was also confirmed by the emergence of cleaved poly (ADP-ribose) polymerase (PARP). Meanwhile, the expression of antiapoptotic Bcl-2 and phosphorylation of prosurvival Akt were shown to be attenuated. Finally, autophagy apparently contribute to cell death as increased expression of the autophagosomal marker LC3-II was detected along with deregulated expression of other autophagy-related proteins, including Atg3, Atg5, Atg7, Atg12 and Beclin-1. In general, these findings were more prominent in combination therapy. Similar results were obtained with KATO-III and LS174T cells (data not shown).

## Discussion

Bromelain is a crude, aqueous extract from the pineapple comprised of sulfhydryl-containing proteolytic enzymes as the main constituents, and a variety of non-proteolytic enzymes and components [10,11]. Being available as a pharmaceutical product since 1956 [12], bromelain has shown benefits in digestion, wound healing, burn debridement, and enhancement of antibiotic absorption. Immunomodulatory, anti-coagulative and anti-inflammatory activities of bromelain with potential applications in various pathological conditions, including infections, cardiovascular and respiratory diseases and musculoskeletal injuries, have been reported [13,14]. Moreover, anticancer properties of bromelain have been shown in different studies [15]. N-acetylcysteine (NAC), known as a sulfhydryl group donor naturally found in Allium plants, is the acetylated derivative of the amino acid L-cysteine and a precursor to the powerful antioxidant glutathione. Initial studies in 1960s and 1970s reported clinical benefits of NAC as an effective mucolytic agent in cystic fibrosis [16] and an antidote for acetaminophen overdose hepatotoxicity [17]. Since then, its utility in different pathological conditions, e.g. chronic obstructive pulmonary disease, contrast-induced nephropathy, cardiovascular diseases, neuropsychiatric disorders and cancer, has been investigated [18,19]. Both drugs are considered as inexpensive, well-tolerated agents with good safety profiles.

We found in the present study that bromelain and NAC exert cytotoxic effects on a panel of human gastrointestinal cancer cells of different origin, chemosensitivity and phenotype. MKN45 and KATO-III are gastric cancer cell lines classified as poorly differentiated and signet ring carcinoma cells, respectively. HT29-5F12 and HT29-5M21 are two subpopulations of HT29 colon adenocarcinoma cell line resistant to 5-fluorouracil and methotrexate, respectively. LS174T is a colon adenocarcinoma cell line with a goblet cell-like phenotype. Furthermore, apart from the unified expression of the membrane-associated mucin MUC1, these cells exhibit different expression phenotypes with respect to the gastrointestinal secretory mucins. While MKN45 and KATO-III both represent a predominantly gastric phenotype and specifically express MUC5AC [20], HT29-5F12 and HT29-5M21 mainly express MUC2 and MUC5AC, respectively [21], and LS174T expresses the goblet cell-specific mucins MUC2, MUC5AC and MUC6 [22].

Anticancer properties of bromelain or NAC, individually, have been evaluated in some *in vitro* and *in vivo* models before. Bromelain has shown cytotoxic and/or cytostatic effects on murine lung carcinoma, mammary adenocarcinoma, leukemia, lymphoma, sarcoma, melanoma and ascitic tumor cell lines [23-26], as well as on human cell lines derived from gastric and colon carcinoma [23,27,28], glioma [29], breast cancer [30-32], epidermoid carcinoma, melanoma [33] and malignant peritoneal mesothelioma [34]. Bromelain was also found to induce differentiation of leukemia cell lines *in vitro* [35] and to exert chemopreventive effects on skin [36-38] and colon [28] tumorigenesis *in vivo*. Clinically, however, benefits of bromelain in cancer have been explored in few studies [39-42]. NAC has been reported to inhibit growth, proliferation and/or invasive behavior of human cancer cells, including colorectal [43], bladder [44,45], prostate [46], tongue [47] and lung [48] carcinoma cell lines, *in vitro*. In addition, anticarcinogenic [49,50] and chemoprotective [51] properties of NAC and its potential value as a chemopreventive agent [52-55] or a chemoprotectant [56-61] has been investigated. NAC has also been shown to interact with and enhance cytotoxic effects of chemotherapeutic drugs [62,63], interferon α [64], copper [65] and epigallocatechin-3-gallate [66] on cancer cells. In contrast, few contradictory reports are also available. Tysnes et al. [29] observed that bromelain significantly and reversibly reduced adhesion, migration and invasion of glioma cells, but did not affect cell viability *in vitro*. In a recent study by Sceneay et al. [67], while NAC targeted hypoxic response of breast cancer cells *in vitro*, it did not inhibit, but even enhanced, tumor growth *in vivo*. Together, these findings imply that bromelain and NAC might function in a cell- and/or context-dependent manner. Examining the efficacy of the two agents in combination, we interestingly found in the present study the synergistic and additive interactions between bromelain and NAC with resultant potentiation of cytotoxicity in combination therapy. Here, we report for the first time cytotoxic effects of bromelain and NAC on gastrointestinal cancer cells with added value in combination therapy.

Mechanistically, we found that bromelain and NAC, in particular in combination, inhibit cell cycle progression and induce both apoptotic and autophagic cell death in the gastrointestinal carcinoma cells. Orderly progression of the cell cycle from one phase to another is coordinated by cyclins sequentially activating their partner proteins, cyclin-dependent kinases (CDKs) [68]. This is initiated by the expression of cyclin D in early G1 which drives the cell cycle through to late G1 and followed by subsequent induction of cyclin E, cyclin A and cyclin B at late G1, early S and late S phases, respectively. Thus, our results indicating diminution of cyclins D, A and B suggest that treatment halts cell cycle progression in early G1. This was found to be associated with activation of intrinsic and extrinsic caspase-dependent apoptotic pathways and inhibition of pro-survival pathways which collectively reduce cell viability. Finally, autophagy was shown to apparently contribute to cell death when deregulation of autophagy-related proteins and, more importantly, an increase in the expression of LC3-II, known as the most reliable marker of autophagosomes [69], were observed. Mechanistic basis for the growth-inhibitory effects of bromelain or NAC on malignant cells have been explored in a number of studies. Kalra et al. and Bhui et al. have demonstrated in their investigations on mouse skin tumors that bromelain treatment is associated with upregulation of p53 and Bax, activation of caspase 3 and caspase 9, attenuation of Erk and Akt phosphorylation and decrease in Bcl-2 [37,38]. They later reported that bromelain treatment of human epidermoid carcinoma and melanoma cells, *in vitro*, resulted in cell cycle arrest at G(2)/M phase (by modulation of cyclin B1, phospho-cdc25C, Plk1, phospho-cdc2 and myt1) and subsequent induction of apoptosis through modulation of Bax-Bcl-2 ratio, apoptotic protease activating factor 1 (Apaf-1), caspase-9, and caspase-3 [33]. In a separate study on breast cancer cells *in vitro*, they demonstrated induction of apoptosis and autophagy in response to bromelain where autophagy preceded and facilitated apoptotic response [30]. Bromelain-induced apoptosis in breast [31,32] and colon cancer cells [28] has also been supported by other investigators.

As reviewed by De Flora et al. [70], NAC utilizes a variety of mechanisms to inhibit carcinogenesis. NAC has been shown to inhibit 12-O-tetradecanoylphorbol-13-acetate (TPA)-mediated induction of cyclin D1 and DNA synthesis [71], to diminish DNA synthesis in human astrocytoma cells [72], to inhibit p38 mitogen-activated protein kinase (MAPK) activation and abnormal cell cycle progression in human lymphoma cells [73], and to induce CDK inhibitors and G1 cell cycle arrest in murine papilloma cells [74]. Moreover, despite a large body of evidence establishing anti-apoptotic effects of NAC protecting normal cells against cytotoxic stimuli, there are reports arguing for a pro-apoptotic role of NAC. As an initial observation, Tsai et al. [75] reported that NAC induce apoptosis in aortic smooth muscle cells but not in endothelial cells. In agreement, Liu et al. [76] and Havre et al. [77] indicated that NAC selectively induced p53-mediated apoptosis in several oncogenically-transformed fibroblasts, but not in normal cells. In a study by Nargi et al. [43], it was demonstrated in a range of colorectal cancer (CRC) cell lines that NAC differentially induced cell cycle arrest or apoptosis in CRC cells and identified CDK inhibitor p21(WAF1/Cip1), functional p53, Ras status and basal levels of reactive oxygen species (ROS) in CRC cells as important determinants of susceptibility to apoptosis. Cho et al. also found that NAC reduced ROS production and Akt phosphorylation in breast cancer cells, resulting in apoptotic cell death [78]. NAC has also been shown to enhance H_2_O_2_-, UV- and MK886-induced apoptosis of murine hybridoma [79], human melanoma [80] and human T-cell leukemia cells [81], respectively. These reports and our findings conclusively support the notion that NAC can exert pro-apoptotic effects in a cell type- and context-dependent manner.

## Conclusion

In summary, we found in the present study that bromelain and NAC, on their own and more potently in combination, inhibit growth, proliferation and survival of human gastrointestinal cancer cells of different phenotypes and characteristics. Our data suggests that bromelain in combination with NAC could be of potential value in novel therapeutical approaches to cancer. With special regard to peritoneal malignancies and carcinomatosis, this formulation holds promise for enhancement of microscopic cytoreduction in locoregional strategies and thus represents an interesting avenue for future research.

## Abbreviations

Apaf-1: Apoptotic protease activating factor 1
CDKs: Cyclin-dependent kinases
CI: Combination index
CRC: Colorectal cancer
DMF: Dimethylformamide
GAPDH: Glyceraldehyde 3-phosphate dehydrogenase
IC_50_: Fifty percent inhibitory concentration
MAPK: Mitogen-activated protein kinase
NAC: N-acetylcysteine
NEAA: Non-essential amino acids
PARP: Poly (ADP-ribose) polymerase
PBS: Phosphate buffered saline
RIPA: Radio-Immunoprecipitation assay
ROS: Reactive oxygen species
rTdT: Recombinant terminal deoxynucleotidyl transferase
SSC: Saline-sodium citrate
TPA: 12-O-tetradecanoylphorbol-13-acetate
TUNEL: Terminal deoxynucleotidyl transferase (TdT)-mediated deoxyuridine triphosphate (dUTP) nick-end labeling
